# Data-Enhanced Deep Greedy Optimization Algorithm for the On-Demand Inverse Design of TMDC-Cavity Heterojunctions

**DOI:** 10.3390/nano12172976

**Published:** 2022-08-28

**Authors:** Zeyu Zhao, Jie You, Jun Zhang, Yuhua Tang

**Affiliations:** 1 State Key Laboratory of High Performance Computing, College of Computer, National University of Defense Technology, Changsha 410073, China; 2 Defense Innovation Institute, Academy of Military Sciences PLA China, Beijing 100071, China

**Keywords:** inverse design, transition metal dichalcogenides, photonic cavity, integrated heterojunction, strong coupling effect, deep learning, data enhancement, reinforcement learning

## Abstract

A data-enhanced deep greedy optimization (DEDGO) algorithm is proposed to achieve the efficient and on-demand inverse design of multiple transition metal dichalcogenides (TMDC)-photonic cavity-integrated heterojunctions operating in the strong coupling regime. Precisely, five types of photonic cavities with different geometrical parameters are employed to alter the optical properties of monolayer TMDC, aiming at discovering new and intriguing physics associated with the strong coupling effect. Notably, the traditional rigorous coupled wave analysis (RCWA) approach is utilized to generate a relatively small training dataset for the DEDGO algorithm. Importantly, one remarkable feature of DEDGO is the integration the decision theory of reinforcement learning, which remedies the deficiencies of previous research that focused more on modeling over decision making, increasing the success rate of inverse prediction. Specifically, an iterative optimization strategy, namely, deep greedy optimization, is implemented to improve the performance. In addition, a data enhancement method is also employed in DEDGO to address the dependence on a large amount of training data. The accuracy and effectiveness of the DEDGO algorithm are confirmed to be much higher than those of the random forest algorithm and deep neural network, making possible the replacement of the time-consuming conventional scanning optimization method with the DEDGO algorithm. This research thoroughly describes the universality, interpretability, and excellent performance of the DEDGO algorithm in exploring the underlying physics of TMDC-cavity heterojunctions, laying the foundations for the on-demand inverse design of low-dimensional material-based nano-devices.

## 1. Introduction

One of the most significant current research topics in low-dimensional materials is transition metal chalcogenides (TMDC) due to their attractive and alluring physical properties [1], strong exciton oscillator strength associated with a large binding energy [2], and evident valley degree of freedom [3]. These provide TMDCs with a great potential for versatile applications in light emitting, light detection, and optical modulation [4,5,6]. Take monolayer (ML) WS_2_ for instance, which not only owns a direct bandgap and an exciton binding energy of ∼0.71 eV but also demonstrates large optical absorption, high quantum yields and a strong photoluminescence (PL) effect in the visible regime [7,8]. In practice, it is not feasible to directly use the optical properties of ML WS_2_ in photonic devices considering the giant nonradiative decay rates. Fortunately, the photonic cavity is viewed as one of the promising platforms that can easily tune and engineer local optical density states of TMDCs after their integration by means of the coupling effect between the cavity mode and TMDC excitons [9,10], which greatly enhances the luminescence and absorption characteristics [7]. Especially when operating in the strong coupling regime [11], new half-light-half-matter quasi-particles denoted as polaritons are formed, along with the Rabi splitting behavior [12], causing a highly concentrated light field at the interface. Moreover, the strong coupling effect can trigger tremendous novel physics phenomena in the TMDC-cavity heterojunctions [13,14] and enrich the functionalities for polariton devices, such as polariton lasers [15], transistors [16], and logic circuits [17]. 

In fact, there have been a growing number of publications focusing on the room temperature polaritons in ML TMDC-based heterojunctions since 2017. Three independent research groups led by A. Tartakovskii [18], N. Stern [19], and V. Menon [20], respectively, reported their experimental observation of exciton-polariton in the distributed Bragg reflection (DBR) optical microcavity and ML TMDCs-integrated heterojunctions at room temperature, demonstrating an exciting and significantly large valley degree of freedom. Other previous works were performed at low temperatures with a corresponding valley degree of freedom between 10% and 30% [21]. Room temperature operation is the prerequisite for practical applications of such devices in novel quantum computing, communications, and detection. Normally, the spin energy valley of TMDCs excitons does not show up at room temperature, but it is realizable to observe such phenomenon when TMDC excitons are coherently coupled with micro-cavity photons. Importantly, a variety of photonic cavities have been employed in the exciton-polariton studies [22,23], whose process often requires either complicated experimental measurements or conventional numerical simulations that consume expensive computing resources. Therefore, an intelligent and powerful prototyping tool that can not only characterize the optical response of TMDC-cavity heterojunctions but also realize the on-demand inverse design of such devices is highly desired.

Recently, tremendous literature has witnessed a prominent development of deep learning (DL) algorithms, a significant branch of machine learning which has been utilized as an attractive and widespread method in scientific research considering its giant advances in computer technology. The DL algorithms are employed in various fields, such as nature language processing [24,25,26], image recognition [27], finance [28,29,30], and medicine [31,32,33]. Especially for the aspect of nanophotonics, the DL approach has proved to be one of the most advanced tools to study many nonintuitive and nonlinear physics issues [34,35,36,37], including the problem of the inverse design of photonics devices [38,39,40,41,42,43,44,45]. Peurifpy et al. [46] utilized 50,000 samples to train neural networks to design a multi-layer dielectric spherical nanoparticle in 2018, which was regarded as the landmark in this field. Raju et al. [47] proved the practicality of DL by fabricating plasmonic patterns designed by the algorithm on the prepared nanolaminate in 2022. All these studies demonstrate the apparent advantages of DL algorithms in terms of accuracy and speed, which enable the conduction of the devices’ inverse design in a precise and much faster way compared to frequently used conventional scanning methods. However, traditional DL algorithms are confronted with two problems. Firstly, most traditional DL schemes often rely on accurate modeling of the problem to make an appropriate prediction directly and neglect a decision-making strategy. Here, “directly” in the inverse design problem means that, by inputting the target spectra into the model, it can predict the corresponding geometry parameters in just one step. Secondly, the DL model training process is usually highly data-dependent, but generating a large dataset in conventional numerical simulations is extremely time- and resource-consuming.

**Figure 1:**
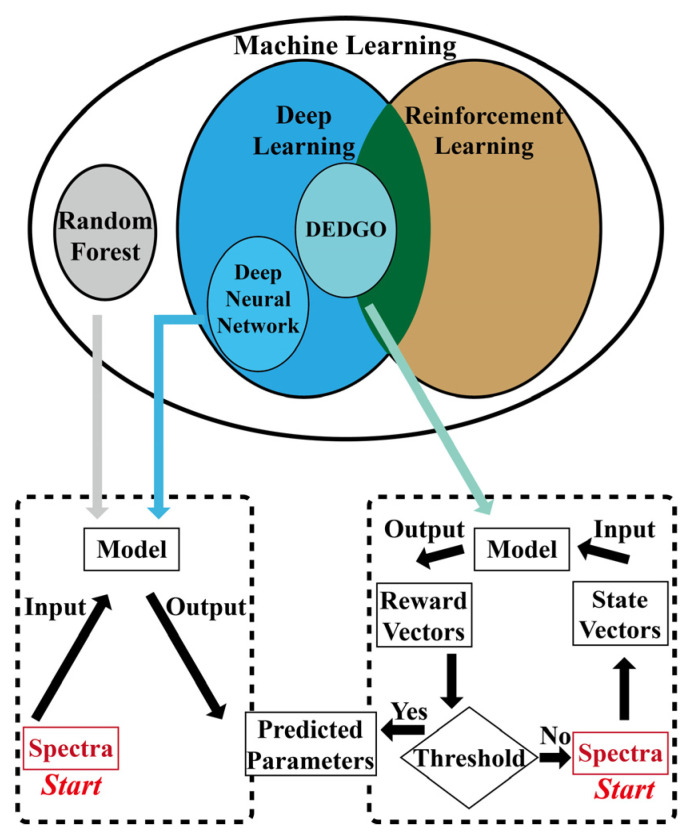
Principles and operating modes of different algorithms. The ellipses show their principles, and the boxes reveal their operating modes, direct prediction (the left box), and iterative optimization (the right box).

To address the above two issues, a data-enhanced deep greedy optimization (DEDGO) algorithm is proposed, with two remarkable features. One is DEEP GREEDY OPTIMIZATION, using the “reward function network” to iteratively accumulate improvements and optimize the predictions rather than using a monolithic network to directly solve the problem, which demonstrates significant advantages over the random forest (RF) and deep neural network (DNN). The models’ differences in terms of principles and operating modes are schematically shown in Figure 1. As for the principle, the DEDGO algorithm constitutes the combination of DL and reinforcement learning (RL), while DNN only belongs to DL, and RF belongs to machine learning. In terms of operating mode, RF and DNN belong to direct prediction methods, while the DEDGO algorithm is an iterative optimization method. The iterative optimization method mainly relies on the “reward function network” (i.e., “Model” in the right box). It optimizes the prediction of geometric parameters according to the Reward Vectors (i.e., the output of the model). If the spectral error is less than the threshold, the optimization is completed. Otherwise, the new spectra will compose new State Vectors and continue the iteration process. This strategy realizes the inverse design through automatic and intelligent iterations, improving the performance and robustness of the model. The effect of this strategy cannot be achieved through iterations using the direct prediction methods, since their predictions remain unchanged when the target spectrum is constant. The design inspiration of the “reward function network” mainly comes from the “reward function” in RL. Retaining some RL factors that are conducive to solving the optimization problem in a local and greedy way is a wise choice, considering the difficulties in training an RL model with high suitability. Here, “greedy” means that the algorithm only makes the most beneficial choice for the current rather than the overall situation. The other pivot is DATA ENHANCEMENT (DE), targeting enriching the dataset using “pseudo data” to train large networks with small amounts of simulated data, decreasing the time consumption in spectroscopic simulations, and improving the prediction performance of the algorithm. The “pseudo data” are generated statically, which guarantees the denoising and filtering for it before utilization, reducing the systematical errors introduced by data enhancement. Especially, the “pseudo data” should be distinguished from the “original data” generated by the traditional rigorous coupled wave analysis (RCWA) method.

Concretely, The DEDGO algorithm is applied to handle the inverse design problem of TMDC-cavity heterojunctions with a three-dimensional parameter space, namely, the incident angle *θ*, the structural variable *g*, and whether the photonic cavity is to be integrated with the monolayer WS_2_. By utilizing the rigorous coupled wave analysis (RCWA) approach and the DEDGO algorithm, the reflection spectra of multi-shaped heterojunctions with geometrical parameters are studied. The process of the algorithm can be separated into two phases, namely, the training that focuses on data enhancement and network training and the testing that utilizes the reward function model to optimize the solution greedily step by step. Importantly, comprehensive results confirm that the DEDGO algorithm is a powerful and promising tool that can not only achieve the efficient inverse design for multiple heterojunctions and the exploration of the underlying physics regarding strong coupling effects in a precise (>85% effectiveness) and rapid manner (several seconds for 89 testing samples) but also has obvious advantages over traditional scanning optimization methods and many other machine learning methods in terms of universality, accuracy, and time consumption.

## 2. Materials and Methods

### 2.1. Heterojunctions with Different Structures

**Figure 2:**
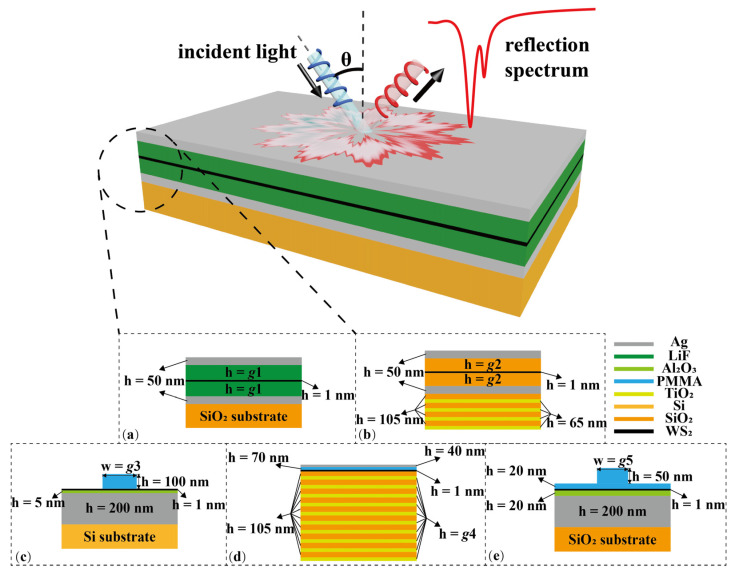
Schematic of different WS_2_-photonic cavity-integrated heterojunctions. Precisely, the top figure illustrates the light–matter interaction when the heterojunctions are irradiated by an incident light with an angle of *θ*, while the bottom figures represent the heterojunction structures of S1 (**a**), S2 (**b**), S3 (**c**), S4 (**d**), and S5 (**e**), respectively.

The main aim of this work is to achieve the on-demand design of cavity-TMDC-based heterojunctions utilizing relatively few reflection spectra generated by traditional RCWA simulations. More specifically, the schematic illustration of the monolayer (ML) WS_2_-photonic cavity-integrated heterojunction is presented in Figure 2. One can easily discern the light–matter interaction between the heterojunction and an incident light with an incident angle of *θ*. Herein, five different categories of cavity-TMDC-based heterojunctions are studied in this work, whose geometrical structures are shown in Figure 2a–e, respectively: (1) Figure 2a represents a Fabry–Perot (FP) microcavity containing two silver mirrors and an LiF integrated with ML WS_2_ that is inserted in the middle of the LiF layer, whose hybrid structure is referred to as S1. (2) The WS_2_-based heterojunction denoted as S2 is illustrated in Figure 2b, whose cavity is similar to the S1 cavity, with the structural difference being the distributed Bragg reflector (DBR) consisting of four layers of SiO_2_ and TiO_2_. (3) Figure 2c shows a S3 heterojunction possessing ML WS_2_ that is sandwiched between a one-dimensional (1D) periodically PMMA photonic crystal (PhC) and a passivated silver substrate. (4) The S4 heterojunction comprising ML WS_2_ and a Tamm-plasmon cavity is displayed in Figure 2d, in which the top mirror is a 40 nm silver film and the bottom mirror is a 10-layer DBR (SiO_2_ and TiO_2_ pairs). (5) Figure 2e shows a S5 heterojunction whose structure is akin to S3 but with waveguide type changes from strip to rib in 1D PhC. It is significant to emphasize that altering the geometrical parameters of photonic cavities allows for the control and engineering of cavity photonic modes, which then affect the coupling effect between the cavity and ML WS_2_. Thus, we also change the parameter *g* of microcavities in S1–S5 heterojunctions, namely, the half-thickness of the LiF film in S1 (*g*1: 80–92 nm), the half-thickness of the SiO_2_ film in S2 (*g*2: 74–86 nm), the width of the PMMA strip waveguide in S3 (*g*3: 160–400 nm), the thickness of the TiO_2_ layer in S4 (*g*4: 59–71 nm), and the width of the PMMA rib waveguide in S5 (*g*5: 40–160 nm). For convenience, the thickness of the ML WS_2_ film used in these structures is fixed to 1 nm, whilst in cases of pure photonic cavities, the thickness of ML WS_2_ is automatically set to 0 nm in numerical simulations. In addition to the structural variable *g*, the incident angle is also an important factor for the inverse design, which varies between −40 degrees and 40 degrees.

As a matter of fact, the incident angle *θ* is measured in degrees, and the structural variable *g* is measured in nanometers, accompanied by the different variation ranges of *g*1–*g*5, making it impossible to directly apply these datasets in the DEDGO training process. Therefore, a normalization method is highly desired to make the above parameters be on the same scale. Thus, the parameter normalization method can be represented as [45]:

(1) $$x_{\mathrm{nor\_angle}}=\frac{x-x_{\min}}{\delta_{\mathrm{angle}}}$$

(2) $$x_{\mathrm{nor\_g}}=\frac{x-x_{\min}}{\delta_{g}}+1$$

where *x* represents the actual value, *x_min_* stands for the minimum value, and *δ* is the distance of discrete attributes. Notably, the dataset contains only reflectance spectra with non-negative incident angles are included in the dataset due to the symmetry feature, with the incident angle resolution being 1°. Here, the structural variable *g* is regulated to be positive integers varying between 1 and 13 after normalization, whereas the thickness of ML WS_2_ can only be selected between 0 and 1 for pure photonic cavity and TMDC-cavity heterojunctions, respectively. 

The RCWA method is a fundamental finite element analysis method to characterize the interaction between electromagnetic waves and nanostructures, which is specifically tailored for multilayer structures. Here, the RCWA approach is employed to calculate the reflection response of S1–S5 heterojunctions and their corresponding pure cavities, generating 298 reflection spectra serving as the training dataset for the DEDGO algorithm, with a wavelength range of 500–900 nm and an incident angle change from −40~40 degrees. Notably, the whole simulation procedure of RCWA covers the structure model design, the optical properties implementation for each material, the boundary condition determination, the convergence test, and the scanning simulation. The original datasets are divided into three parts, which are used in the control and experimental groups in different proportions: (1) 57%, 13%, and 30% for the training, validation, and testing in control groups (without data enhancement); (2) 70%, 0%, and 30% for the training, validation, and testing in experimental groups (with data enhancement). The validation datasets of experimental groups are composed of “pseudo data” calculated via data enhancement.

### 2.2. Data-Enhanced Deep Greedy Optimization Algorithm

**Figure 3:**
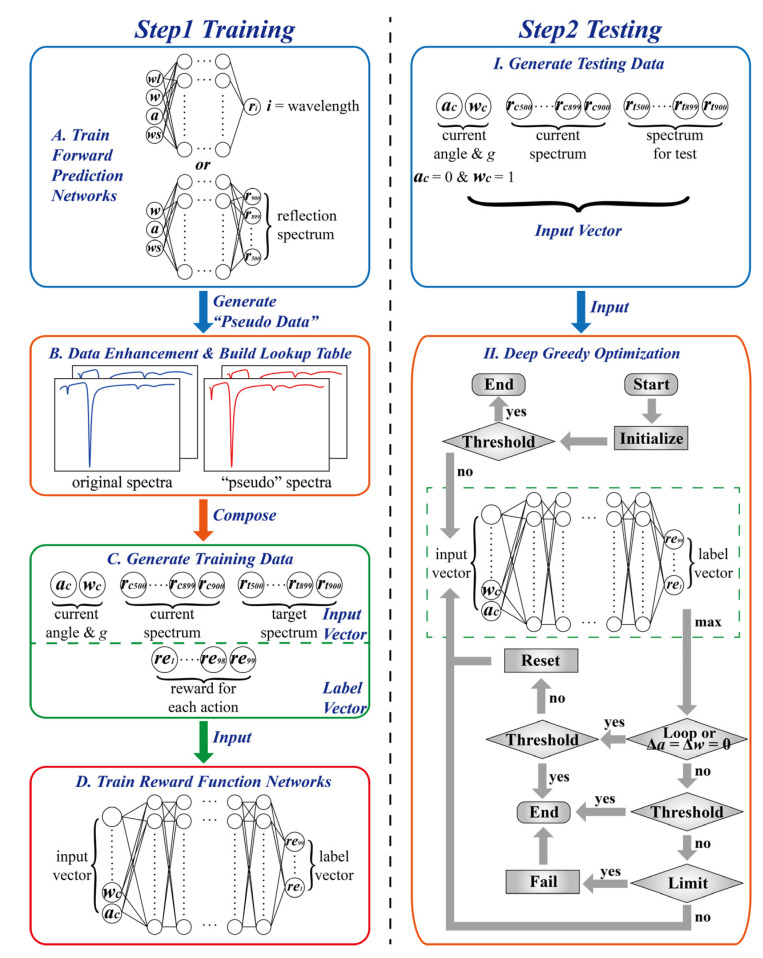
Schematics of the DEDGO algorithm for the efficient inverse design of multiple heterojunctions. The algorithm can be divided into two main stages, namely, training (**left**) and testing (**right**). The first stage is applying the forward prediction networks to intensify the dataset (**A**,**B**) and training the reward function networks for the next part (**C**,**D**). The second part is utilizing the trained reward function networks to predict structural parameters for heterojunctions in a gradual optimization way (**I**,**II**).

The working flow of the DEDGO algorithm is shown in Figure 3, which contains two main modules. The first module is the forward prediction networks, trained for data enhancement. The second module is the reward function networks, trained for deep greedy optimization, which acquire angle and structural variable parameters step by step and distinguish previous deep learning methods that attempt to obtain the structural parameter value directly. The essential innovation of the DEDGO algorithm lies in the data source and in the usage of reward function networks. In particular, the data enhancement method can rapidly expand datasets and easily meet the data requirements of deep learning methods at a low cost, improving the fitting level of the model to complex nonlinear relationships between parameters and reflection spectra. On the other hand, the deep greedy optimization can realize higher accurate predictions of parameters in an acceptable speed using a simple network compared to other machine learning approaches. This is mainly due to its “iteration” and “reset” mechanism, which can reduce the model training difficulty by avoiding predicting parameters directly. 

The exhaustive process of training is described as follows: first, a neural network for forward predictions with seven hidden layers is trained with the original training spectra. Notably, this work uses two slightly different network structures for the S1–S5 heterojunctions to predict their reflection spectra precisely. The network takes the angle and geometrical parameters as its input and the spectra indicating the wavelength from 500 nm to 900 nm as its output. Furthermore, data enhancement based on this model has shown a high reliability. Since the absolute values of reflection spectra usually vary between 0 and 1, it is necessary to convert the spectra into a space with greater absolute values in order to make the network extraction much easier [34]:

(3) y′=−100 lg y

where y represents the original spectra and y′ denotes the values after calculation. The loss function of the forward prediction network is described by the mean absolute error (MAE) [37]:

(4) $${\mathrm{Loss}}_{\mathrm{forward}}\;=\;\frac{1}{n}\overset{n}{\underset{i=1}{\Sigma }}\mathrm{abs}\left(y_{\mathrm{pred}}^{’i}-y_{\mathrm{true}}^{’i}\right)$$

where *n* is the batch size, y’_pred_ represents the prediction of the spectra, and y’_true_ is the corresponding true reflection spectra. The MAE of the results is in the 10^−2^ order of magnitude, indicating the credibility of the predicted spectra. In this instance, approximately 750 reflection spectra are generated within 0.06 s to intensify the dataset as “pseudo” spectra. The angle *θ* and structural variable *g* combinations from the “pseudo” spectra are different from the spectra created via RCWA simulations. The “pseudo” spectra are added to the training and validation datasets in a 1:1 proportion. Note that “pseudo” spectra are not used to test the model to avoid possible spectral errors. Additionally, the lookup table is established to speed up the subsequent step, producing training data for reward function networks.

**Figure 4:**
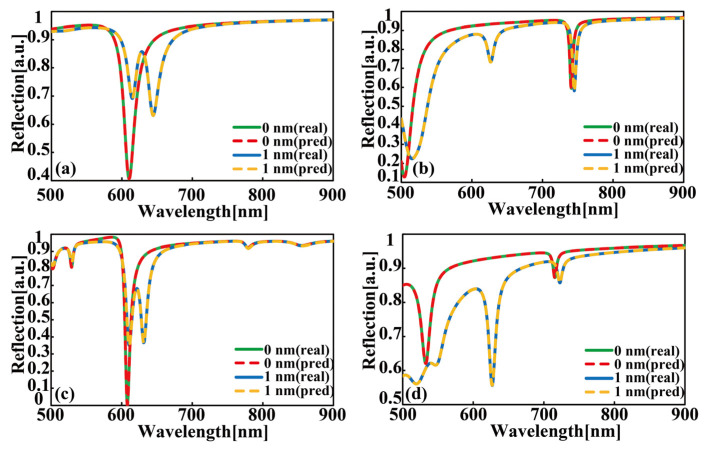
Comparison of the reflection spectra calculated by the RCWA simulation (real, solid lines) and forward prediction network (pred, dotted lines). (**a**–**d**) The reflection spectra incident at 20 degrees with different WS_2_ thicknesses for S1, S3, S4, and S5 from top to bottom, respectively. Concretely, the values of structural variable *g* are given as follows: S1: LiF, 85 nm; S3: PMMA, 22 nm; S4: TiO_2_, 65 nm; S5: PMMA, 100 nm.

To validate the effectiveness of the forward prediction network in the DEDGO model, we illustrate the comparison results of the reflection spectra calculated by the RCWA method and the forward prediction network for the S1, S3, S4, and S5 heterojunctions and their pure cavity counterparts in Figure 4. Note that the reflection spectra of S1 and S2 are highly similar, so we only display the reflectance result of S1 in this figure. Here, we randomly select the incident angle of reflection spectra to be 20°and fixed geometrical parameters for four categories of samples. Specifically, the values of structural variable *g* are given as follows: S1: LiF, 85 nm; S3: PMMA, 22 nm; S4: TiO_2_, 65 nm; S5: PMMA, 100 nm. One distinctive finding from Figure 4 is that the DEDGO-predicted results are in extremely good agreement with the RCWA-simulated spectra, indicating the dependability and high accuracy of the DEDGO network using “pseudo data”. Furthermore, it can be easily extracted from Figure 4 that, after the integration of ML WS_2_ with the photonic cavity, extra reflectance resonances occur in the spectra, in contrast to the original one main resonant peak, indicating the physical phenomena of Rabi splitting and the strong coupling effect.

There are distinct differences between the forward prediction network and the reward function network, which are mainly reflected in the network structure, input, and output. The reward function network is a 5-hidden-layer model, whose input and output are an 804-number and a 99-number vector, respectively. The input vector consists of three parts, namely, the current angle *θ* and structural variable *g*, the current spectrum, and the target spectrum. The current angle *θ* and structural variable *g* are inseparable from the current spectrum. These two elements, together with the target spectrum, make up the input vector called “state”, which contains two aspects of information. Firstly, it embodies the implicit relationship between the incident angle, structural variable, and reflection spectrum. Secondly, it provides an opportunity for the model to extract the diversity between the two spectra, making greedy optimization possible. Here, “optimization” means reducing the discrepancies between the current spectrum and the target spectrum to obtain more precise used parameters by changing the incident angle and structural variable, whose adjustment process is defined as “action”. Moreover, “greedy” indicates that the model selects actions that better reduce the spectral differences, characterized by the usage of mean absolute percentage error (MAPE) [45]:

(5) $$\mathrm{MAPE}=\frac{1}{n}\overset{n}{\underset{i=1}{\sum }}\mathrm{abs}\left(\frac{y_{\mathrm{cur}}^{i}-y_{\mathrm{tar}}^{i}}{y_{\mathrm{tar}}^{i}}\right)\times 100\%$$

Here, “MAPE” is calculated from the target spectrum and the spectrum corresponding to the “current parameters” in the “state vector”. Similarly, it is necessary to quantify the actions in a proper way. Thus, the “reward” for each action can be described as follows:

(6) $$\mathrm{reward}=\left\{\begin{array}{ll} -0.75, & \Delta \mathrm{MAPE}\in (-\infty ,-7.5]\\ 0.1\;\Delta \mathrm{MAPE}, & \Delta \mathrm{MAPE}\in (-7.5,7.5)\\ 0.75, & \Delta \mathrm{MAPE}\in [7.5,\infty ) \end{array}\right.$$

(7) $$\Delta \mathrm{MAPE}\;=\;{\mathrm{MAPE}}_{\mathrm{ori}}-{\mathrm{MAPE}}_{\mathrm{new}}$$

where MAPE_ori_ and MAPE_new_ denote the errors before and after executing the action, respectively. The error between the target spectrum and the spectrum corresponding to new predicted parameters is reduced when “ΔMAPE” is greater than 0. Obviously, the better the action that can narrow the spectral gap, the higher the reward that can be obtained. According to the “greedy” principle, the algorithm will select the “action” with the largest reward to execute. On this condition, the reward of the selected action must be greater than or equal to 0. Notably, the reward equals 0 when the action is “changing the parameters by 0 units”. Therefore, each iteration gains some improvements and optimization. It is almost impossible to optimize the MAPE to zero due to the potential small errors introduced in each step. In the circumstances, the spectral error is small enough when the MAPE is less than 0.25 for functional consideration, and the current incident angle and structural variable can be regarded as proper target parameters. The algorithm sets up 99 actions to achieve this goal as quickly as possible and to minimize the training difficulty of the model. The adjustment range is −5~5 for angle *θ*, which is −4~4 for structural variable *g*. The output vector contains the rewards for these 99 actions, during which the reward function networks need to learn the relationship between actions and rewards in different current spectra and target spectra. 

The theme of the testing stage is to employ the trained model to calculate the corresponding parameters of the spectra. The current angle and structural variable are set to 0 and 1 to ensure the validity and reliability, respectively. This preset corresponds to “Initialize” in Figure 3II. It is indispensable to compare the initial spectrum with the spectrum for the test and calculate their MAPE. If the MAPE is less than the threshold, the sample can be exempted from optimization; thus, 0 and 1 are taken as the true values of the target parameters. Otherwise, the vector is input into the trained reward function network, and the action with the maximum reward is chosen to optimize the vector. After that, the corresponding spectral response can be compared with the target value to calculate the MAPE. In particular, the action is recorded to verify whether the action itself changes the parameters by zero or whether the optimization is in a loop. Specifically, a “loop” means that the total changes of the two parameters by several actions are both zero. The occurrence of these two cases implies that the process is stuck in a local optimum. If the MAPE is less than the current threshold, the inverse design is still successful. Otherwise, it is requisite to reset the input vector to guarantee that the optimization can continue normally. If the algorithm does not get stuck in the local extrema and the MAPE is less than the threshold, the current parameters can be seen as target parameters. In another case with a lager MAPE, the algorithm checks the number of steps that have been run. If it exceeds the limit, the algorithm declares a failure; otherwise, the optimization process continues. The limit is set to 30 in this research considering the balance of time and effect. 

The mentioned neural network in the DEDGO algorithm is coded using an open-source artificial intelligent framework, namely, TensorFlow2, and run on an RTX 3090 graphic card and an i9-10900X CPU. The Adam optimization algorithm is chosen as the optimizer to train the networks.

## 3. Results and Discussion 

### 3.1. Inverse Design of the Heterojunctions

**Figure 5:**
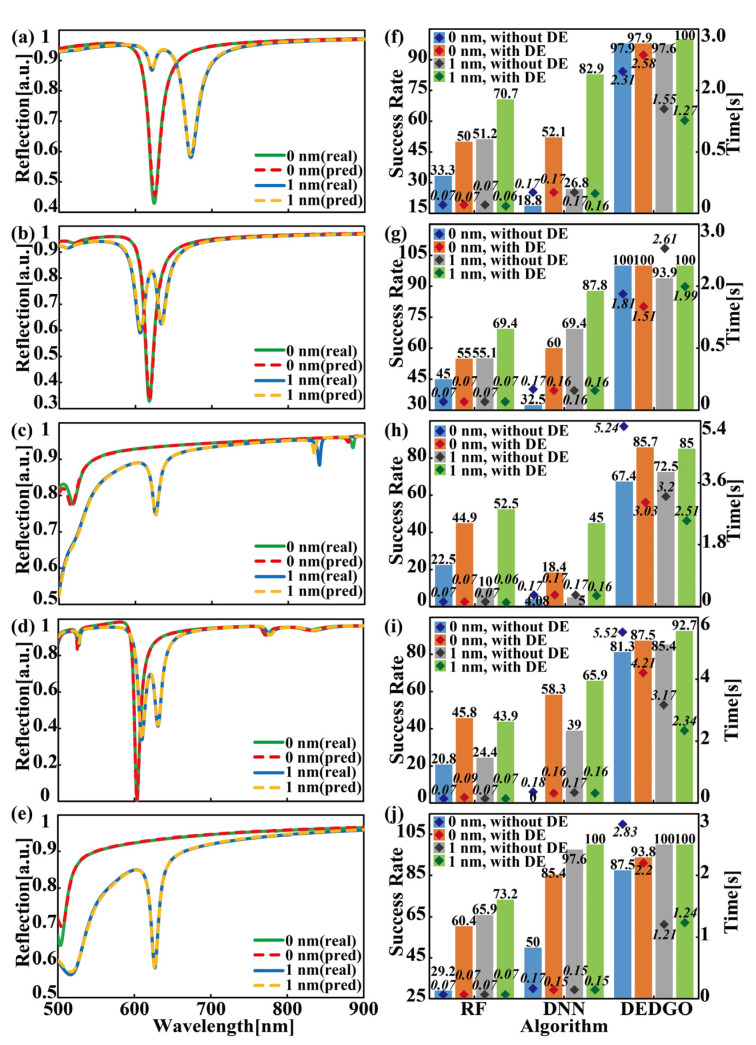
Typical results of inverse design with the DEDGO algorithm. (**a**–**e**) Spectral comparison of true (real, solid lines) and predicted values (pred, dotted lines) for S1–S5 heterojunctions (1 nm) and their pure cavity counterparts (0 nm). (**f**–**j**) The predicted success rate and time consumption for samples in different categories based on the DEDGO algorithm. (**a**–**e**) Concretely, the MAPE of the spectra are shown as follows: S1: 0 nm, 0.22; 1 nm, 0.23; S2: 0 nm, 0.24; 1 nm, 0.22; S3: 0 nm, 0.18; 1 nm, 0.25; S4: 0 nm, 0.22; 1 nm, 0.24; S5: 0 nm, 0.21; 1 nm, 0.24. Notably, the 0 nm thickness for the WS_2_ film indicates the case of a pure photonic cavity, unless otherwise specified.

In this section, the DEDGO algorithm is utilized to achieve the efficient and on-demand inverse design of TMDC-cavity-based heterojunctions, intending to signify the model’s superiority. For comparison, RF and DNN algorithms are also applied in the testing stage, indicating the effectiveness of the DEDGO algorithm in inverse design. More specifically, RF uses the original spectra as its input, since processing the spectra according to Equation (3) has little or even negative effects on this method. DNN employs the same hidden layer structure as the reward function network for a better comparison. In particular, the above two methods attempt to directly predict the parameters of the 401-bit testing spectra, which can, however, be regarded as an acceptable prediction in the case where the MAPE of the testing spectrum and the spectrum corresponding to predicted parameters is less than the threshold. The testing dataset for each heterojunction is 89 randomly selected samples from the original spectra composed of 298 items. The number of samples with different WS_2_ thicknesses varies slightly, because sampling is conducted with a total number criterion. The typical results of inverse design with the DEDGO algorithm are shown in Figure 5. To demonstrate the reasonableness of the threshold selection and the effectiveness of the DEDGO algorithm, the spectral comparison of true and predicted values for the S1–S5 heterojunctions (1 nm) and their pure cavity counterparts (0 nm) is shown as Figure 5a–e, revealing that the inverse design results can satisfy the optical functional demands when the error is less than the threshold. The reliability of this conclusion is guaranteed by the randomly selected 10 sets of geometrical parameters. Notably, we present the original reflectance spectra calculated via RCWA and the spectra of inverse-designed low-dimensional structures for a vivid comparison. One significant finding from the left panels of Figure 5 is that the relevant inverse design results are consistent with the input data, indicating the strong and powerful inverse design ability of DEDGO. Furthermore, the quantified design results are shown in Figure 5f–j, which can be divided into four groups according to the thickness of WS_2_ and whether data enhancement is used.

Two indicators, namely, the success rate and computing time, are chosen to describe the performances of the three algorithms. Here, “success rate” refers to the proportion of predictions that hit the true value accurately or whose spectral errors are less than the threshold, revealing the effectiveness of the algorithm. “Time” records the computational time needed to complete the inverse design process on the testing dataset. It can be easily found from Figure 5f–j that the DEDGO algorithm owns a much larger success rate than RF and DNN for all S1–S5 heterojunctions and the corresponding pure cavities. The relative difference is at least 15% to 30%, especially for cases without data enhancement. Furthermore, an at least 85% success rate can be reached by the DEDGO algorithm for all types of samples with data enhancement, revealing its powerful capability. Concretely, the success rate for S1, S2, and S5 is almost 100%, while that for S3 and S4 is slightly lower (85~95%). Note that the S3 group obtains the most performance improvement, while the S5 group gets the least, probably due to the difficulty of feature extraction. As the difficulty increases, the advantages of the DEDGO algorithm outperform the other two algorithms. Nevertheless, this performance improvement comes, in part, at the cost of a larger time consumption due to the two characteristics, namely, “iteration” and “reset”, which introduce extra operations compared with the other two algorithms. The inverse design consumed times of the RF and DNN algorithms are both less than 0.5 s, while it changes from 1.21 to 5.52 s for the DEDGO algorithm. Even though the DEDGO algorithm is a little inferior in this respect, it is still far faster than traditional iterative methods, which pre-configure a parameter combination, calculate the corresponding spectrum, and constantly adjust the parameters until the spectral error is less than the threshold, usually taking tens of minutes or even hours to design a sample [48,49,50]. Therefore, the DEDGO algorithm is confirmed to be an excellent method for the inverse design of TMDC-cavity heterojunctions. Further insight regarding the average success rate and time consumption of the DEDGO, RF, and DNN algorithms is explicitly presented in Table 1.

It is also worthwhile to investigate the role of data enhancement in these machine learning algorithms. Firstly, it is apparent that the data enhancement groups almost always improve the success rate for all of the S1–S5 heterojunctions and their cavity counterparts. This is more pronounced for RF and DNN, whose success rates can even be improved by more than 50%, since their success rate is usually low without data enhancement. In terms of time consumption, data enhancement mainly works on the DEDGO algorithm and has little influence on the other two algorithms, which is probably determined by the running mechanism. As aforementioned, RF and DNN try to predict the parameters directly, which means that more spectral data can only optimize the internal weights and biases of the network rather than the running process of the algorithm, making it difficult to reduce the testing time. However, since the DEDGO algorithm is an iterative method, with the assistance of data enhancement, it can better learn the relationship between parameters, spectra, and rewards to choose better actions, reducing time by decreasing the running steps and restarts. The loss and running performance metrics revealing the effect of data enhancement on the DEDGO algorithm are shown in Table 2. The train loss and validation loss are significantly reduced in the data enhancement groups, suggesting that the network can more accurately capture features and learn abstract relationships. In addition, the effect of fewer running steps and restarts is more pronounced in groups with a lower success rate, such as S3 and S4, which also have the highest drop in testing time. In contrast, this effect is not apparent for groups with a higher success rate due to the randomness of the neural network training process. For instance, the randomness causes more samples with more running steps or restarts for S1 with no WS2, resulting in larger time consumption, although the average steps and restarts are decreased. To conclude, data enhancement makes a remarkable contribution to the DEDGO algorithm, which is an indispensable part, as well as deep greedy optimization.

Besides its high success rate and low time consumption, the universality of the DEDGO algorithm deserves to be mentioned. In the research, five types of heterojunctions and the corresponding photonic cavities are studied, showing the potential of the algorithm to design more different structures. It has a remarkable superiority over most deep learning methods used before.

### 3.2. DEDGO Assisted the In-Depth Study of Multiple Cavity-TMDC Heterojunctions

**Figure 6:**
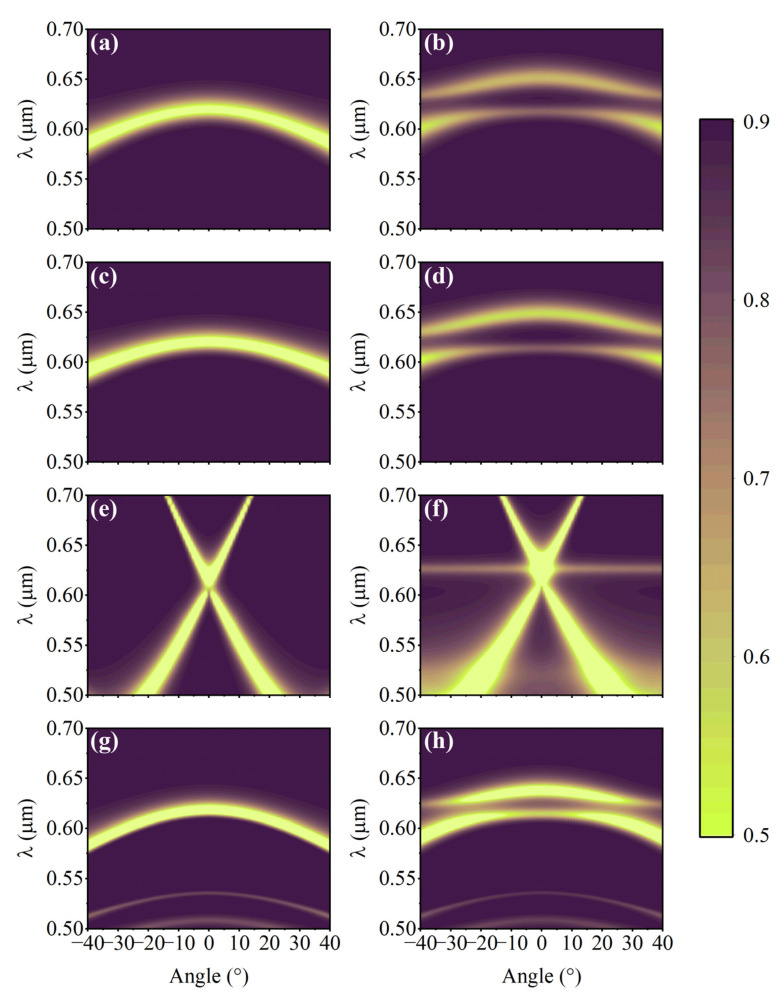
Compared results of angle-resolved reflectance spectra between the individual photonic cavities (**left**) and the cavity-ML WS_2_-integrated heterostructures. (**a**–**h**) From top to bottom, the panels represent the categories of S1 (**a**,**b**), S2 (**c**,**d**), S3 (**e**,**f**), and S4 (**g**,**h**), respectively.

By means of the DEDGO algorithm, we can go further to investigate the nonintuitive and complex relationships between the structural parameters of TMDC-cavity-based heterojunctions and their optical properties. As aforementioned, the geometry, material component, and relevant geometrical parameters are important and intriguing parameters for photonic cavities that dramatically change the light–matter interactions of TMDC-cavity-based heterojunctions. Therefore, to obtain further insight into the coupling effect in the WS_2_-cavity hybrid device, we employ the DEDGO network to characterize and measure the angle-resolved white-light reflectance spectra of S1–S4 heterojunctions; the results are shown in Figure 6. Considering the geometric similarity between the S3 and S5 heterojunctions, the reflectance spectra of S5 are not included in Figure 6. For comparison, we have also included the wavelength–angle dispersions of pure photonic cavities in the left panels of Figure 6 for the cases of TE polarization. It is worthwhile to mention that all the angle-resolved reflectance calculated in this work is performed with an angular resolution of 1° and at room temperature.

The most significant and eye-catching finding from Figure 6 is that the optical-guided modes of photonic cavities strongly overlap with the A exciton of ML WS_2_, illustrated as the horizontal line lying around 0.625 μm in Figure 6b,d,f,h, for all S1–S4 hybrid devices. If one looks deeper into the above results, it is not difficult to observe the clear and apparent anticrossing behaviors associated with progressive dispersions of optical-guided modes through the A excitonic transition, revealing that the hybrid WS_2_-cavity device operates in the strong coupling regime. In other words, the dispersion of guided modes in S1–S4 heterojunctions splits into two parts, which are denoted as the lower and upper polariton bands, indicating the formation of half-light-half-matter quasi-particles. Evidently, the optical guide modes of photonic cavities in S1, S2, and S4 are quite similar since they share the F-P cavity structure, which is distinct from the 1D PhC cavity. However, due to the different parameter selections for the photonic cavity, namely, *g*1 = 85 nm for the S1 cavity, *g*2 = 80 nm for the S2 cavity, *g*3 = 220 nm for the S3 cavity, and *g*4 = 65 nm for the S4 cavity, the corresponding anticrossing points locate at different incident light angles. Another significant conclusion suggested by Figure 6 is that, after the integration of the ML WS_2_ film in the photonic cavities, their optical mode dispersion curves exhibit the red-shift phenomena. Take S3 in Figure 6e,f, for instance—the original mode crossing points locate the 1D PhC cavity around 0.6 μm, which shift to around 0.61 μm after placing WS_2_ underneath 1D PMMA PhC. One reasonable explanation for this effect is the change in the refractive index of the WS_2_-PhC heterojunction compared to pure PhC, where the dielectric environment dominates the optical mode dispersion of the photonic cavity.

## 4. Conclusions

In summary, a DEDGO algorithm based on deep greedy optimization and data enhancement has been established. This algorithm can fulfil the inverse design of multiple TMDC-cavity heterojunctions in a rapid and precise way. With the aid of the RCWA method, a relatively smaller training dataset is generated for the training process of DEDGO, followed by a supplementary dataset created by the integral forward network. Next, the enhanced dataset is employed to train the reward network, which played a major role in achieving the deep greedy optimization strategy in the testing stage. The experiments show that the DEDGO algorithm achieves a higher accuracy than the RF and DNN methods at the cost of a little time. Furthermore, a detailed analysis of the coupling effect between the TMDC excitons and micro-cavity photons in TMDC-based heterojunctions comprising different cavity categories, material components, and geometrical parameters, as well as the on-demand inverse designs, was conducted using the DEDGO algorithm, in which the strong coupling effects and exciton-polaritons were thoroughly revealed. This work shows the outstanding universality and accuracy of the DEDGO algorithm, indicating its excellent potential in the inverse design of multi-shaped heterojunctions and the fabrication of photonic crystals.

## Figures and Tables

**Table 1:** The average success rate and time consumption of different algorithms.

Group	RF		DNN		DEDGO	
	Success Rate	Time Consumption	Success Rate	Time Consumption	Success Rate	Time Consumption
WS_2_ thickness = 0 nm, without DE	30.16	0.07 s	21.08	0.17 s	86.82	3.54 s
WS_2_ thickness = 0 nm, with DE	51.22	0.07 s	54.84	0.16 s	92.98	2.71 s
WS_2_ thickness = 1 nm, without DE	41.32	0.07 s	47.56	0.16 s	89.88	2.35 s
WS_2_ thickness = 1 nm, with DE	61.94	0.07 s	76.32	0.16 s	95.54	1.87 s

**Table 2:** Loss and running performance metrics of the DEDGO algorithm.

Group	Train Loss of Reward Function Networks (MAE)		Validation Loss of Reward Function Networks (MAE)		Average Running Steps of Successful Samples		Restarts on the Testing Dataset	
	With DE	Without DE	With DE	Without DE	With DE	Without DE	With DE	Without DE
S1, WS_2_ thickness = 0 nm	0.0015	0.0040	0.0017	0.0046	6.57	6.65	62	65
S1, WS_2_ thickness = 1 nm	0.00096	0.0022	0.0010	0.0028	5.15	5.44	10	23
S2, WS_2_ thickness = 0 nm	0.0020	0.0048	0.0022	0.0060	5.00	6.55	35	61
S2, WS_2_ thickness = 1 nm	0.00086	0.0023	0.00097	0.0032	5.88	6.63	30	54
S3, WS_2_ thickness = 0 nm	0.013	0.015	0.032	0.12	3.68	5.84	75	154
S3, WS_2_ thickness = 1 nm	0.011	0.014	0.019	0.048	4.76	4.93	67	87
S4, WS_2_ thickness = 0 nm	0.0094	0.016	0.015	0.047	10.45	13.62	88	158
S4, WS_2_ thickness = 1 nm	0.0027	0.0060	0.0030	0.0073	6.74	7.46	68	119
S5, WS_2_ thickness = 0 nm	0.0011	0.0025	0.0015	0.014	5.11	6.34	47	42
S5, WS_2_ thickness = 1 nm	0.0010	0.0020	0.0013	0.0033	4.85	4.71	2	10

## Data Availability

The data presented in this study are available on request from the corresponding author.
